# Optimization design of two-stage amplification micro-drive system without additional motion based on particle swarm optimization algorithm

**DOI:** 10.1186/s42492-022-00124-1

**Published:** 2022-11-25

**Authors:** Manzhi Yang, Kaiyang Wei, Chuanwei Zhang, Dandan Liu, Yizhi Yang, Feiyan Han, Shuanfeng Zhao

**Affiliations:** 1 School of Mechanical Engineering, Xi’an University of Science and Technology, Xi’an, 710054 China; 2 Department of Mechanical and Aerospace Engineering, Brunel University London, Uxbridge, 361199 UK; 3 School of Humanities and Foreign Languages, Xi’an University of Science and Technology, Xi’an, 710054 China

**Keywords:** Particle swarm optimization, Micro-drive mechanism, Two-stage amplification, Optimization design, Performance of guidance

## Abstract

With the increasing requirements of precision mechanical systems in electronic packaging, ultra-precision machining, biomedicine and other high-tech fields, it is necessary to study a precision two-stage amplification micro-drive system that can safely provide high precision and a large amplification ratio. In view of the disadvantages of the current two-stage amplification and micro-drive system, such as poor security, low motion accuracy and limited amplification ratio, an optimization design of a precise symmetrical two-stage amplification micro-drive system was completed in this study, and its related performance was studied. Based on the guiding principle of the flexure hinge, a two-stage amplification micro-drive mechanism with no parasitic motion or non-motion direction force was designed. In addition, the structure optimization design of the mechanism was completed using the particle swarm optimization algorithm, which increased the amplification ratio of the mechanism from 5 to 18 times. A precise symmetrical two-stage amplification system was designed using a piezoelectric ceramic actuator and two-stage amplification micro-drive mechanism as the micro-driver and actuator, respectively. The driving, strength, and motion performances of the system were subsequently studied. The results showed that the driving linearity of the system was high, the strength satisfied the design requirements, the motion amplification ratio was high and the motion accuracy was high (relative error was 5.31%). The research in this study can promote the optimization of micro-drive systems.

## Introduction

As biomedicine, aerospace, microelectronics and other high-precision technical fields have increasingly stringent requirements for precision positioning and micro-drive systems, precision and ultra-precision machining technologies have become an important way to realize the processing of precision machinery and equipment [1–5]. Precisely amplifying and transmitting tiny input displacements has become a research hotspot in the field of precise positioning and micro-drive system positioning. The flexible hinge has the advantages of no friction, no hysteresis, high precision, and no assembly error; the micro-drive mechanism [6–10] uses the flexible hinge guide, transmission and conversion functions to input deformation to provide accurate displacement. The precise amplification of the micro-drive displacement based on flexible hinge technology can satisfy the requirements of motion stroke in the current work [11–13]. However, the amplification ratio of the one-stage amplification micro-drive system is limited, and the provided amplification displacement is small. Therefore, it is of great significance to research two-stage amplification of the micro-drive mechanism.

The parasitic motion and non-motion directional forces of the two-stage amplification micro-drive mechanism affect the precision and safety of the motion of the micro-drive system. Muralidhara and Rao [14] designed a hydraulic displacement amplification system based on the micro drive principle of piezoelectric actuators, which can accurately estimate the displacement behavior within the error range. Li et al. [15] designed an electrostatic micro-actuator with high frequency and chip size compatible with an endoscope microscope with a large vertical scanning range to help generate a large range of motion in such mirrors. Iqbal et al. [16] designed an amplification mechanism based on the displacement amplification mechanism of a micro-electro-mechanical system (MEMS), which can be used independently or in combination with other compatible mechanisms to amplify displacement. However, the two-stage amplification micro-drive structures designed in the above studies do not consider the guiding performance of the mechanism; and cannot ensure that the system has no parasitic motion or non-motion directional forces.

At the same time, the optimization of the motion amplification ratio of the two-stage amplification system can enable the system to obtain the maximum output displacement based on the original output displacement. The mechanism designed by Iqbal et al. [17] based on the displacement amplification mechanism of MEMS can amplify the input displacement 6.8 times. Fan et al. [18] proposed a symmetrical flexure hinge displacement amplification mechanism based on the differential lever principle and established a mathematical model of the amplification ratio, in which the maximum amplification ratio of the mechanism was obtained as 6.50. There are few studies related to the optimization of the amplification ratio of the two-stage amplification system, and the amplification ratio is small; therefore, research on the optimization of the amplification ratio of the two-stage amplification system is of great value.

In summary, a two-stage amplifying micro-drive mechanism was designed using the lever and triangle principles in this study. The mechanism has a symmetrical guiding mechanism, which can eliminate parasitic motion and non-motion directional forces, and ensure the precision and safety of system motion. The mechanism was optimally designed with a certain plane space as the constraint condition to maximize the amplification ratio using particle swarm optimization (PSO, the amplification ratio can reach 18), which can achieve the maximum output displacement and compensate for a larger range of motion. A piezoelectric ceramic actuator (PZT) and two-stage amplifying micro-drive mechanism are used as micro-actuators and actuators, respectively, to design a precision two-stage amplifying micro-drive system. In addition, the driving, strength, and motion performances of the system were analyzed. The analysis results show that the system has excellent correlation performance.

## Methods

### Design of two-stage amplification micro-drive system

The flexible hinge has the advantages of no friction, no hysteresis, high precision, and no assembly error, which can realize the functions of displacement guidance, transmission, and conversion. The main amplification methods of the micro-drive amplification mechanism based on flexible hinge design include lever amplification, triangle amplification, compression rod instability amplification, and special mechanism amplification [19–22]. Among them, the lever amplification mechanism has the advantages of a simple structure, high amplification ratio, and easy processing, while the triangular amplification has a compact structure and excellent dynamic characteristics.

**Fig. 1:**
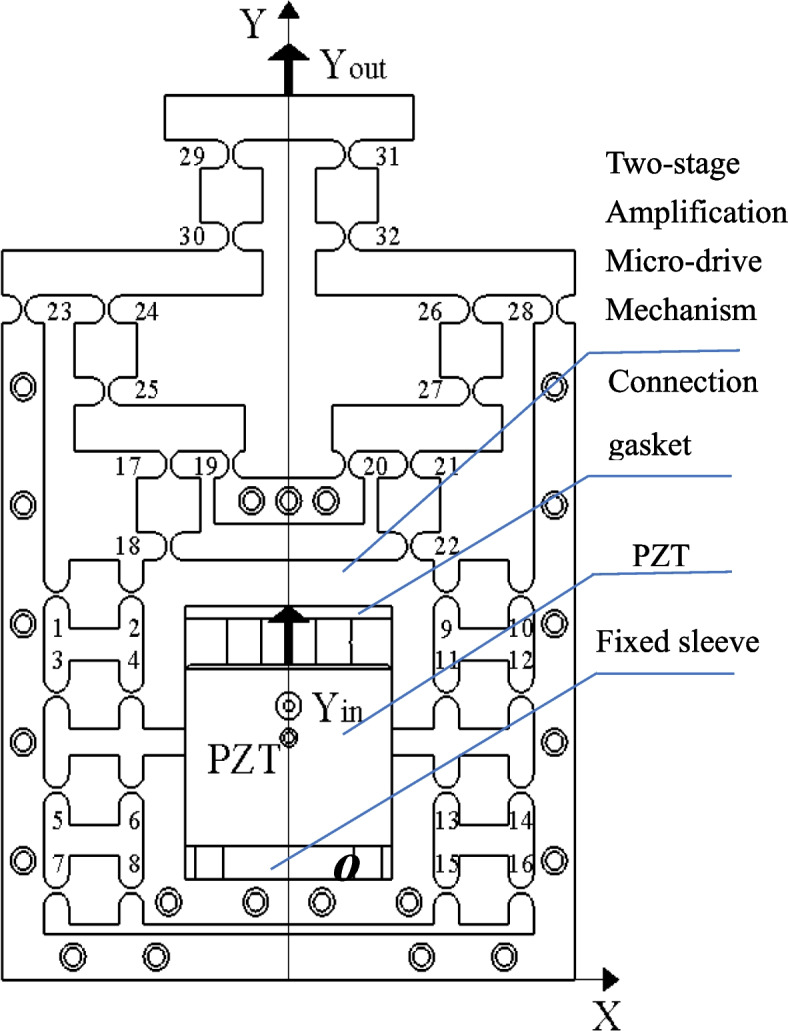
Schematic diagram of working principle of micro-drive system

In this study, a two-stage amplification micro-drive system was designed based on the triangular principle of the flexible hinge. Spring steel 60Si2Mn was selected as the mechanism material. A schematic of the working principle of the micro-drive system is shown in Fig. 1. The overall size of the micro-drive system is 138 × 192 × 50 mm. The micro-drive mechanism was fixed on the worktable with 21 M6 threaded holes and screws, which were driven by piezoelectric ceramic brakes. In addition, 32 straight circular flexible hinges were symmetrically arranged on the micro-drive mechanism, which were used to eliminate parasitic motion and non-motion directional forces to ensure the precision and safety of system motion.

Because the micro-drive system is symmetrically distributed about the Y-axis, the right half is used for the analysis. The two-stage amplification micro-drive system includes a PZT, two-stage amplification micro-drive mechanism, connecting the gasket and fixed sleeve, where a connecting gasket is used to adjust the gap between the PZT and the working surface, and a fixed sleeve is used to limit the position of the PZT. The working process of the system is as follows: The piezoelectric ceramic brake generates an input displacement Y_*in*_, and the input displacement is transmitted to the first-level lever mechanism by flexible hinges 21 and 22, where flexible hinge 20 is fixed. After amplification by the first-level lever, the amplified output displacement is transferred upward by flexible hinges 26 and 27 along the Y-axis to the second-stage lever amplification mechanism. At this time, flexible hinge 28 is fixed. After the second amplification, the displacement is transmitted to the output end by flexible hinges 31 and 32. Finally, the displacement is output at the top of the mechanism. Among them, the 10 holes below flexible hinge 28 are used to fix the mechanism, the 3 holes below flexible hinge 20 can provide a fixing function for the first-level amplifying mechanism, and the 2 holes between flexible hinges 15 are used to fix the mechanism to ensure piezoelectricity. When the ceramic actuator is driven, the system only moves in the positive direction along the y-axis. The bottom two holes of the mechanism are used to fix the mechanism and provide a preload for the PZT when it works.

### Calculation of amplification ratio of micro-drive mechanism

**Fig. 2:**
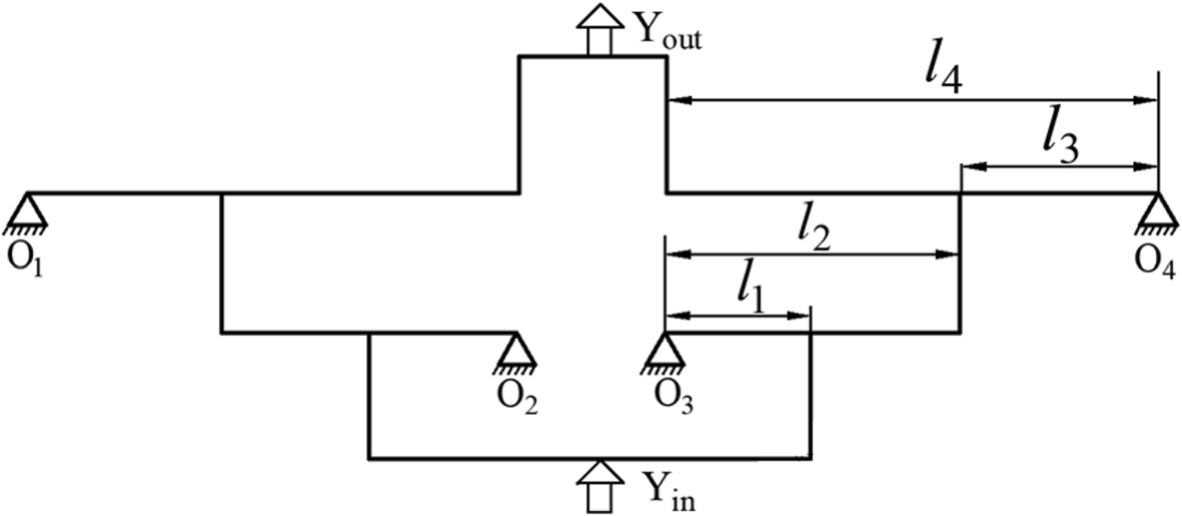
Two-stage amplification principle diagram of micro-drive mechanism

Figure 2 shows a schematic of the amplification of the two-stage amplification micro-drive mechanism. The amplification ratio of this symmetrical two-stage amplification micro-drive mechanism can be adjusted according to the actual needs, and the amplification ratio is changed by adjusting the length of the lever between the flexible hinges. The lengths of *l*_*i*_ (*I* = 1, 2, 3, 4) are shown in the figure.

By analyzing the force and displacement of the first-stage lever, the intermediate transition rod and the second-stage lever in the two-stage amplification micro-drive mechanism, the calculation formula for the amplification ratio of the two-stage micro-drive amplifying mechanism can be expressed as:

1 $$\uplambda =\frac{{\textrm{Y}}_{\textrm{out}}}{{\textrm{Y}}_{\textrm{in}}}=\frac{{\textrm{K}}_{\textrm{M}}^2{\textrm{p}}_1+{\textrm{K}}_{\textrm{M}}{\textrm{K}}_{\textrm{F}}{\textrm{p}}_2+{\textrm{K}}_{\textrm{F}}^2{\textrm{p}}_3}{{\textrm{K}}_{\textrm{M}}^2{\textrm{p}}_4+{\textrm{K}}_{\textrm{M}}{\textrm{K}}_{\textrm{F}}{\textrm{p}}_5+{\textrm{K}}_{\textrm{F}}^2{\textrm{p}}_6}$$

2 $${\textrm{K}}_{\textrm{F}}=\textrm{Eb}{\left(\frac{2\left(2\textrm{s}+1\right)}{\sqrt{4\textrm{s}+1}}\arctan \sqrt{4\textrm{s}+1}-\frac{\uppi}{2}\right)}^{-1}$$

3 $${\textrm{K}}_{\textrm{M}}=\frac{\textrm{EbR}}{12}{\left(\frac{2{\textrm{s}}^3\left(6{\textrm{s}}^2+4\textrm{s}+1\right)}{\left(2\textrm{s}+1\right){\left(4\textrm{s}+1\right)}^2}+\frac{12{\textrm{s}}^4\left(2\textrm{s}+1\right)}{{\left(4\textrm{s}+1\right)}^{\frac{5}{2}}}\arctan \sqrt{4\textrm{s}+1}\right)}^{-1}$$

Where, K_F_ represents axial tensile and compressive stiffness of the flexure hinge; K_M_ represents corner stiffness of the flexure hinge; E represents elastic modulus of the mechanism material; b represents width of the flexure hinge; S represents ratio of cutting radius to minimum thickness of the flexure hinge; ${\textrm{p}}_1=-6{l}_1{l}_4+12{l}_2{l}_4-3{l}_3^2+9{l}_3{l}_4$; ${\textrm{p}}_2=2{l}_1{l}_2\left(4{l}_3{l}_4-3{l}_1{l}_4+6{l}_2{l}_4-{l}_3^2\right)+2{l}_3{l}_4\left(6{l}_2{l}_3-3{l}_1{l}_3-{l}_1^2-2{l}_2\right)$; ${\text{p}}_3=8l_1l_3^2l_4-4l_1^2l_2l_3^2l_4;\;{\text{p}}_4=l_1\left(6l_1-12l_2-19l_3\right)+l_3\left(9l_3+34l_2\right)$;  ${\textrm{p}}_5=4{l}_1{l}_2\left({l}_3^2+5{l}_1{l}_3-3{l}_2{l}_3\right)+2{l}_1{l}_3\left({l}_1{l}_3-4{l}_1^2-6{l}_3^2\right)+4{\textrm{l}}_2{\textrm{l}}_3\left(6{\textrm{l}}_3^2+7{\textrm{l}}_2^2-3{\textrm{l}}_2{\textrm{l}}_3\right)$; ${\textrm{p}}_6=8{\textrm{l}}_1^2{\textrm{l}}_2{\textrm{l}}_3^2-4{\textrm{l}}_1^3{\textrm{l}}_3^3$.

In this two-stage amplification micro-drive mechanism, *l*_1_ = 15 mm, *l*_2_ = 30 mm, *l*_3_ = 20 mm, and *l*_4_ = 50 mm. Substituting into Eq. (1) yields λ = 5.

### Guidance performance analysis of the micro-drive mechanism

The parasitic motion of the micro-drive mechanism with two-stage amplification leads to a decline in the system’s motion accuracy. At the same time, the force in the non-moving direction causes damage to the micro-drive and affects the safety of the system because of the brittle micro-drive of the system in the lateral direction. Therefore, eliminating parasitic motion and force in the non-motion direction during the movement of the mechanism can ensure the precision and safety of the system. In this study, a symmetrical structure is used to realize the guiding function of the mechanism, to ensure that there is no parasitic motion or force in the non-motion direction when the system works.

**Fig. 3:**
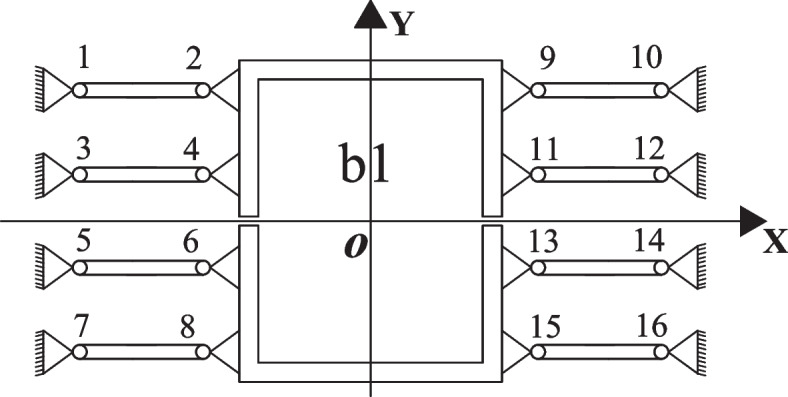
Guiding principle diagram of micro-drive mechanism

The guiding function principle of the two-stage amplification micro-drive mechanism is shown in Fig. 3, The PZT is fixed with b1. During the movement of the two-stage micro-drive amplifying mechanism, 8 flexible hinge components composed of flexible hinges 1 to 16 are designed. During the movement process, the X-direction movement displacement can be eliminated by the flexible hinge deformation of the flexible hinge group, and will not be transmitted to b1, therefore ensuring that the system has no parasitic displacement in the non-motion direction, thereby ensuring the precision of the system motion process.

During the movement, the X-direction force generated by flexible hinges 1–8 and the X-direction force generated by flexible hinges 9–16 are the same in magnitude and opposite in direction, and they cancel out during movement. It does not bear a lateral force or moment, thereby ensuring the safety of the system during movement.

In summary, a symmetrical structure design is adopted in the two-stage amplifying micro-drive mechanism, and the guiding principle of the two-stage micro-drive amplifying mechanism can eliminate the forces in the parasitic motion and non-motion directions, thereby ensuring the precision and safety of the system.

### Driver performance analysis

The PZT is a miniature driving element designed using the inverse piezoelectric effect of piezoelectric materials. Under the action of voltage, the PZT can generate a displacement of several micrometers to tens of micrometers. It can be used for micro-precision driving of the mechanism. Owing to its high resolution, fast response, small size and large output force, it has been increasingly used in the field of precision positioning. The nature of piezoelectric ceramics determines their properties.

A PZT was chosen as the micro-driver for this system, which has shortcomings such as creep, hysteresis, and nonlinearity. These characteristics are related to the properties of piezoelectric materials, such as the electrostrictive effect, piezoelectric and inverse piezoelectric effects, and ferroelectric effects. To investigate the driving performance of the micro-drive system designed in this study, according to the requirements of the system for component size, output force, and output displacement, a PZT (P-235.1 s) was selected from Puai Nano Displacement Technology PI Company, Germany. The maximum displacement of the closed loop was 15 μm. A driving performance test was conduct using this PZT.

**Fig. 4:**
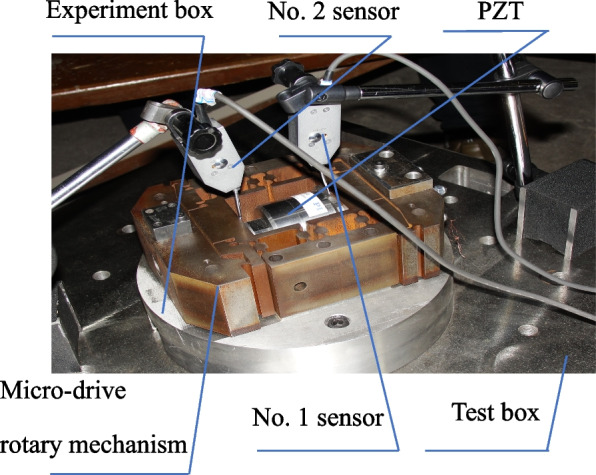
Driving performance experiment diagram of PZT ceramic actuator

The piezoelectric ceramic nonlinear characteristic test is shown in Fig. 4, using the test box, PZT, micro-rotating mechanism, test base, and inductive displacement sensor (the displacement sensor was a bypass inductance with a resolution of 0.05 μm). The test box was placed on the vibration isolation table, the base was fixed on the test box, and the micro-drive rotary mechanism was fixed on the base after assembling the PZT. The voltage U of the PZT is controlled using the control system. The displacement changes of straight inductive sensors No. 1 and No. 2 in the y direction are δy_1_ and δy_2_, respectively, and the elongation of the PZT is:

4 $$\textrm{u}=\left|\updelta {\textrm{y}}_1\right|+\mid \updelta {\textrm{y}}_2\mid$$

**Fig. 5:**
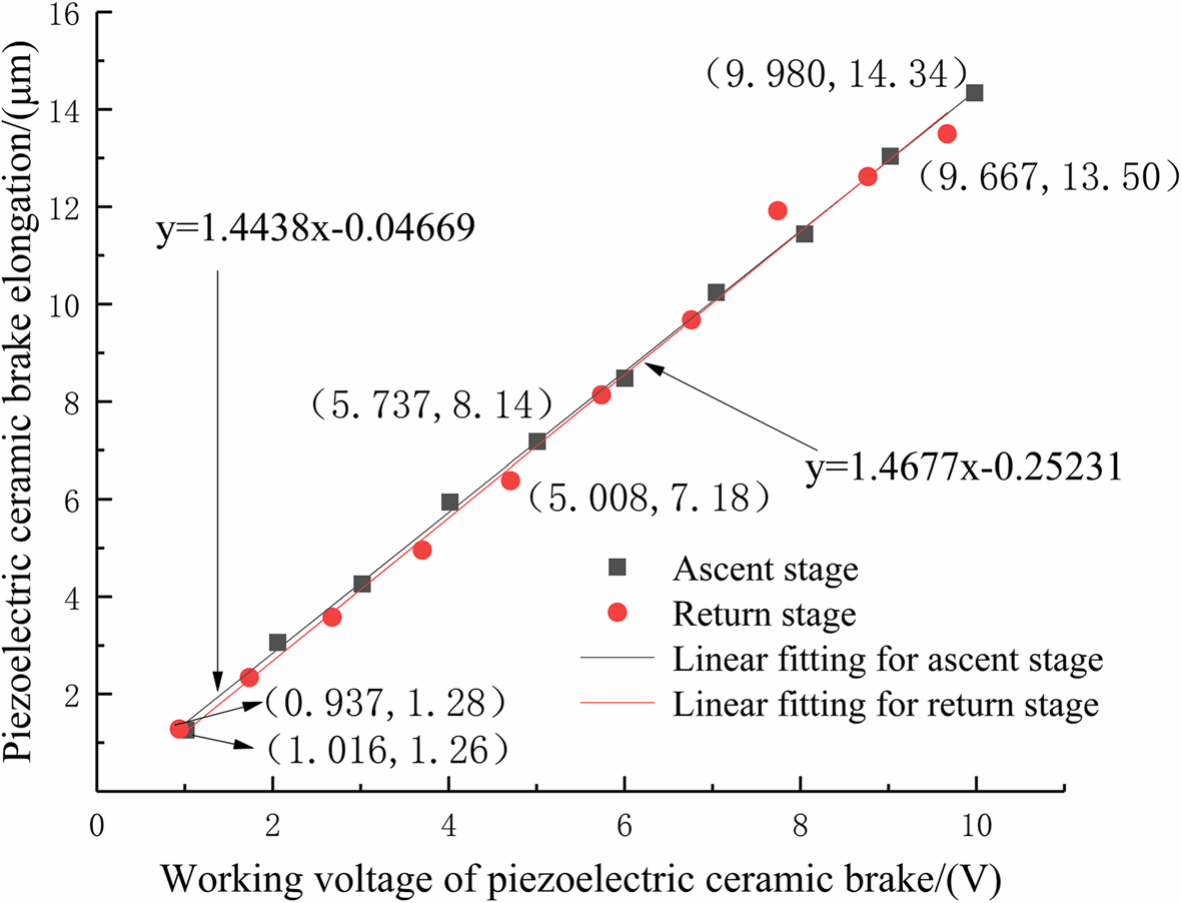
Linear fitting diagram of driving performance of PZT

The ascent and declining motion performances of the PZT were tested. The linear fitting of the test results is shown in Fig. 5.

The working voltage U of the PZT in the ascent stage and the elongation u of the PZT were linearly fitted to a linear equation as follows:

5 $$\textrm{u}=1.4438\textrm{U}-0.04669$$

Its linearity is 0.9992.

The piezoelectric working voltage U and piezoelectric elongation u in the declining phase were linearly fitted to a linear equation as follows:

6 $$\textrm{u}=1.4677\textrm{U}-0.25321$$

Its linearity is 0.9942.

From the test results and linear fitting results, it can be seen that the driving performance of the system is excellent (the maximum error in the ascent and declining stages is only 0.02%), and the minimum linearity is 0.9942.

### Optimization of two-stage amplification micro-drive mechanism

#### Introduction to PSO

To obtain the maximum amplification ratio within a limited space, it is necessary to optimize the structure of the mechanism. PSOs treat each potential solution of the optimization problem as a bird, and the optimal solution is the ‘food’ found by the particle swarm [23–25]. Compared with other algorithms, PSOs have a faster convergence speed, and all particles may converge to the optimal solution more rapidly.

Assuming that an initial population of N particles is generated in a D-dimensional space, the position vector of the ith particle can be expressed as:

7 $${\textrm{x}}_i=\left({\textrm{x}}_{i1},{\textrm{x}}_{i2},{\textrm{x}}_{i3},{\textrm{x}}_{i\textrm{D}}\right),i=1,2,3\cdots \textrm{N}$$

The velocity vector of the ith particle can be expressed as:

8 $${\textrm{v}}_i=\left({\textrm{v}}_{i1},{\textrm{v}}_{i2},{\textrm{v}}_{i3},{\textrm{v}}_{i\textrm{D}}\right),i=1,2,3\cdots \textrm{N}$$

The individual optimal extremum currently searched by the ith particle can be expressed as:

9 $${\textrm{p}}_{\textrm{best}}=\left({\textrm{p}}_{i1},{\textrm{p}}_{i2},{\textrm{p}}_{i3},{\textrm{p}}_{i\textrm{D}}\right),i=1,2,3\cdots \textrm{N}$$

The global optimal extremum currently being searched by the entire population can be expressed as follows:

10 $${\textrm{g}}_{\textrm{best}}=\left({\textrm{g}}_{i1},{\textrm{g}}_{i2},{\textrm{g}}_{i3},{\textrm{g}}_{i\textrm{D}}\right)$$

After the two optimal extreme values are found, the particle updates its speed and position using Eqs. (11) and (12), and finally obtains the optimal solution according to the requirements.

$${\textrm{v}}_{\textrm{i}}\left(\textrm{t}+1\right)=\upomega {\textrm{v}}_{\textrm{i}}\left(\textrm{t}\right)+{\textrm{c}}_1\times \operatorname{rand}\left(\right)\times \left({\textrm{pb}}_{\textrm{id}}\left(\textrm{t}\right)-{\textrm{x}}_{\textrm{i}}\left(\textrm{t}\right)\right)$$

11 $$+{\textrm{c}}_2\times \operatorname{rand}\left(\right)\times \left({\textrm{g}}_{\textrm{bd}}\left(\textrm{t}\right)-{\textrm{x}}_i\left(\textrm{t}\right)\right)\times \left({\textrm{g}}_{\textrm{bd}}\left(\textrm{t}\right)-{\textrm{x}}_i\left(\textrm{t}\right)\right)$$

12 $${\textrm{x}}_{\textrm{i}}\left(\textrm{t}+1\right)={\textrm{x}}_{\textrm{i}}\left(\textrm{t}\right)+{\textrm{v}}_{\textrm{i}}\left(\textrm{t}+1\right)$$

where t is the current iteration number, ω is the inertia factor, rand() is a random number between (0, 1), c_1_ is the individual learning factor, c_2_ is the social learning factor, pb_id_ is the local maximum of particle i in the d-th dimension of the optimal solution, and g_bd_ represents the d-th dimension of the global optimal solution.

#### Structural optimization of the micro-drive mechanism

Because the two-stage amplification micro-drive mechanism is symmetrical about the Y axis, the right half is still used for optimization analysis. As can be seen from Figs. 1 and 2, the completion of the two-stage amplification process is mainly completed by hinges 20, 21, 26, 27, 28, and 32, and the amplification ratio is only related to the distance between the six hinges, and the mechanism is only on the Y-axis. There is displacement in the axial direction; therefore, the optimization goal is transformed into the X-axis coordinate value of the six hinges. The optimization is carried out with hinge 21 as the fixed since better optimization effect was found in this configuration. Therefore, the coordinate value of hinge 20 is set as *x*_1_ (mm), that of hinge 21 is *x*_2_ (mm), that of hinge 27 is *x*_3_ (mm), that of hinge 28 is *x*_4_ (mm), that of hinge 26 is *x*_5_ (mm), and that of hinge 32 is *x*_6_ (mm).

The objective function for this problem is:

13 $$\textrm{K}=\frac{{\textrm{Y}}_{\textrm{out}}}{{\textrm{Y}}_{\textrm{in}}}={\textrm{K}}_1\times {\textrm{K}}_2=\frac{{\textrm{l}}_2}{{\textrm{l}}_1}\times \frac{{\textrm{l}}_4}{{\textrm{l}}_3}=\frac{\left({\textrm{x}}_3-{\textrm{x}}_2\right)\times \left({\textrm{x}}_4-{\textrm{x}}_6\right)}{\left({\textrm{x}}_2-{\textrm{x}}_1\right)\times \left({\textrm{x}}_4-{\textrm{x}}_5\right)}$$

The constraints are:

$$5\le {\textrm{x}}_1<{\textrm{x}}_2,{\textrm{x}}_1<{\textrm{x}}_2<{\textrm{x}}_3,{\textrm{x}}_2<{\textrm{x}}_3<{\textrm{x}}_4,{\textrm{x}}_3<{\textrm{x}}_4\le 65,$$

$${\textrm{x}}_5={\textrm{x}}_3,20\le {\textrm{x}}_6<{\textrm{x}}_5,{\textrm{x}}_2-{\textrm{x}}_1\ge 10,{\textrm{x}}_4-{\textrm{x}}_5\ge 10$$

In addition, *x*_1_, *x*_2_, *x*_3_, *x*_4_, *x*_5_, and *x*_6_ must be integer.

The particle swarm program was written using MATLAB software for the optimization calculation, and the x-coordinates of the new hinge are obtained as:

$${\textrm{x}}_1=5,{\textrm{x}}_2=15,{\textrm{x}}_3=55,{\textrm{x}}_4=65,{\textrm{x}}_5=55,{\textrm{x}}_6=20.$$

By substituting the optimized value into Eq. (13), K = 18 can be obtained. Compared with the previous one, the amplification ratio of the two-stage amplification micro-drive mechanism was significantly improved. Comparing the theoretical calculation value after PSO with the theoretical calculation value before optimization, the structure optimized by PSO can obtain a better solution than the result before optimization under constraints. The amplification ratio of the amplification micro-drive mechanism is increased from 5 to 18 times, and the theoretical movement stroke is 3.6 times the original.

**Fig. 6:**
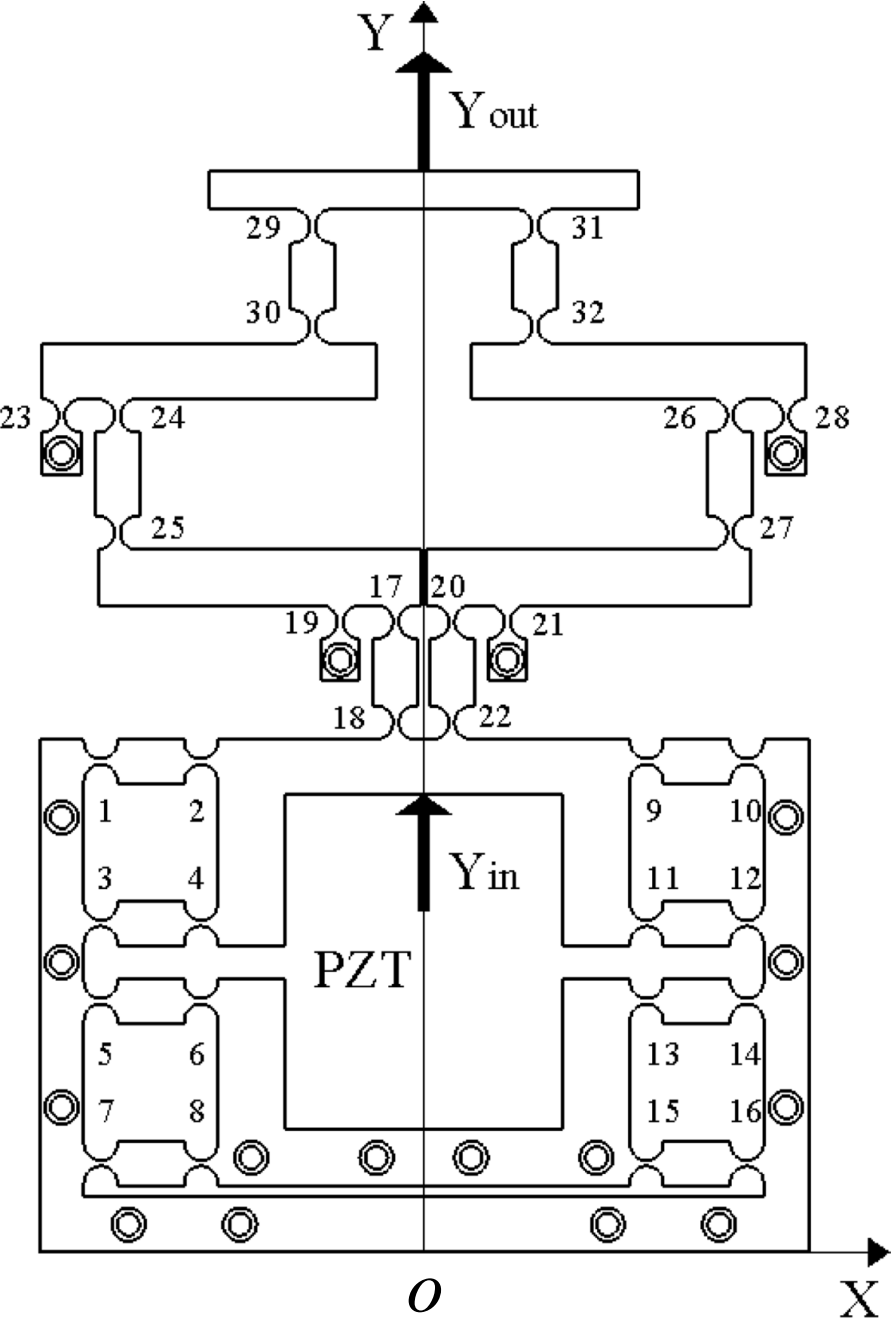
Working principle diagram of two-stage amplification micro-drive mechanism optimized

The working principle diagram of the optimized two-stage magnifying micro-drive mechanism is shown in Fig. 6, which is similar to the working principle of the mechanism shown in Fig. 1 before optimization, uses the flexible hinge triangle principle and lever principle. When the input displacement is Y_in_, the two-stage amplification output displacement Y_out_ is obtained under the action of flexible hinges 20, 21, 26, 27, 28, and 32. In the new structure, flexible hinges 21 and 28 were used as fixed ends, flexible hinges 20 and 27 were used as the input ends of the first-level amplification and two-stage amplification of the two-stage amplification micro-drive mechanism, respectively, and flexible hinges 27 and 32 were used as the two-stage amplification. The output ends of the first-level amplification and two-stage amplification of the amplification micro-drive mechanism were used. The 8 flexible hinge components composed of flexible hinges 1–16 are used to balance the additional force in the non-moving direction and eliminate the parasitic motion and force in the non-moving direction. Spring steel 60Si2Mn was selected as the material for the micro-drive mechanism.

## Results and discussion

### Analysis of strength performance

The strength performance of a system is an important factor affecting its safety. In this study, the finite element method was used to analyze the strength performance of the micro-drive system.

**Fig. 7:**
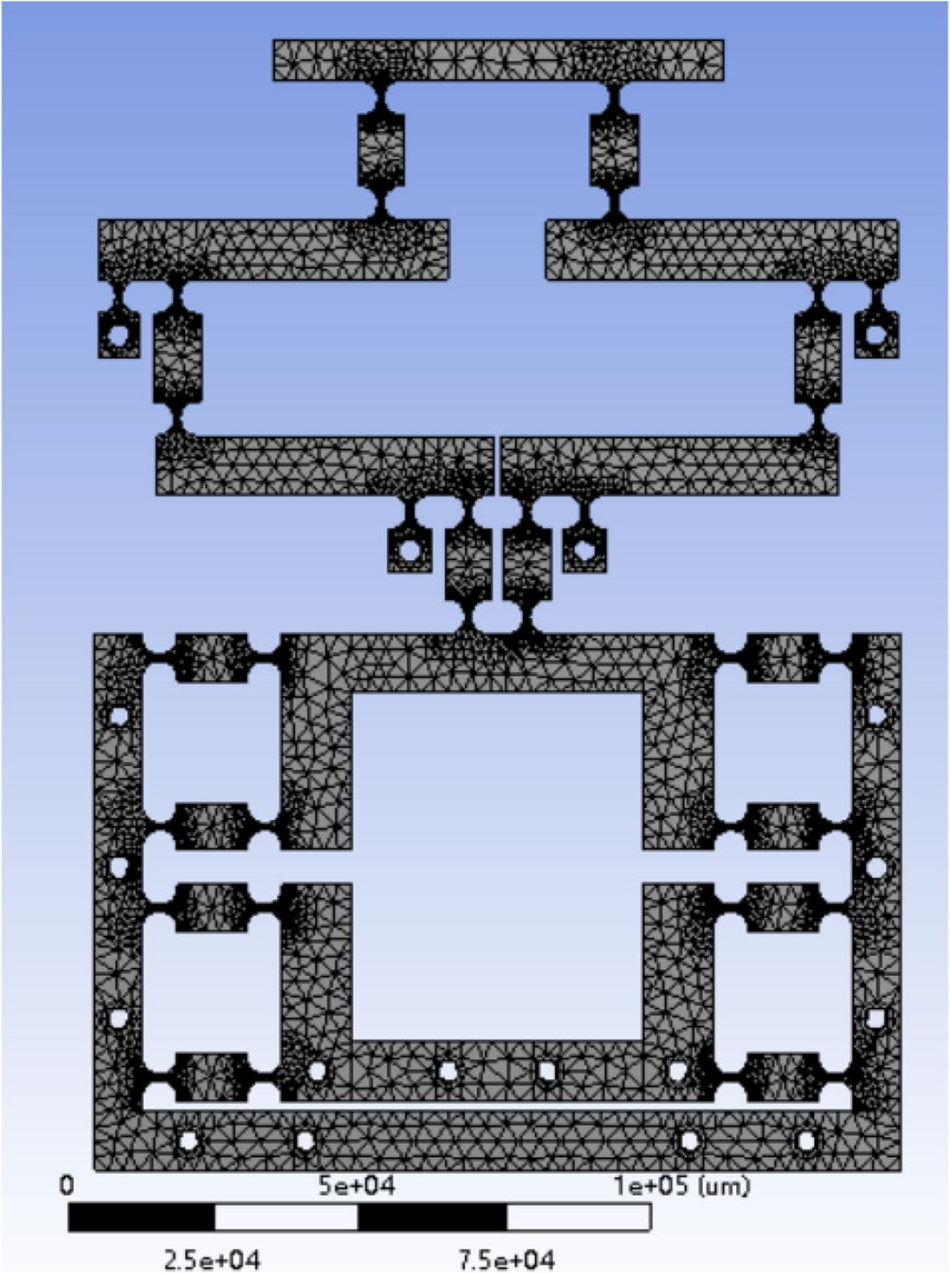
Meshing diagram of micro-drive mechanism

The two-stage amplification micro-drive mechanism drawn in the Solidworks software is saved using the “. x_t ” format, and is then imported into the finite element statics module to generate and press the fixed contact surface between the model and the PZT. The Electroceramic actuators contact surface imprints with the same shape to achieve loading. Using a finite element static analysis, 60Si2Mn parameters were selected for the material properties. During mesh division, the entire model was first meshed freely as a whole, and then, semi-circular radians of 64 flexure hinges from 32 flexure hinges were selected for refinement of the mesh cells with parameter refinement to 1. A grid diagram of the partitioned mechanism is presented in Fig. 7. The model was divided into 390,140 units and 612,361 nodes. It can be seen from the figure that the meshing quality is excellent as the key parts are relatively fine and smooth without cross or broken meshes.

**Fig. 8:**
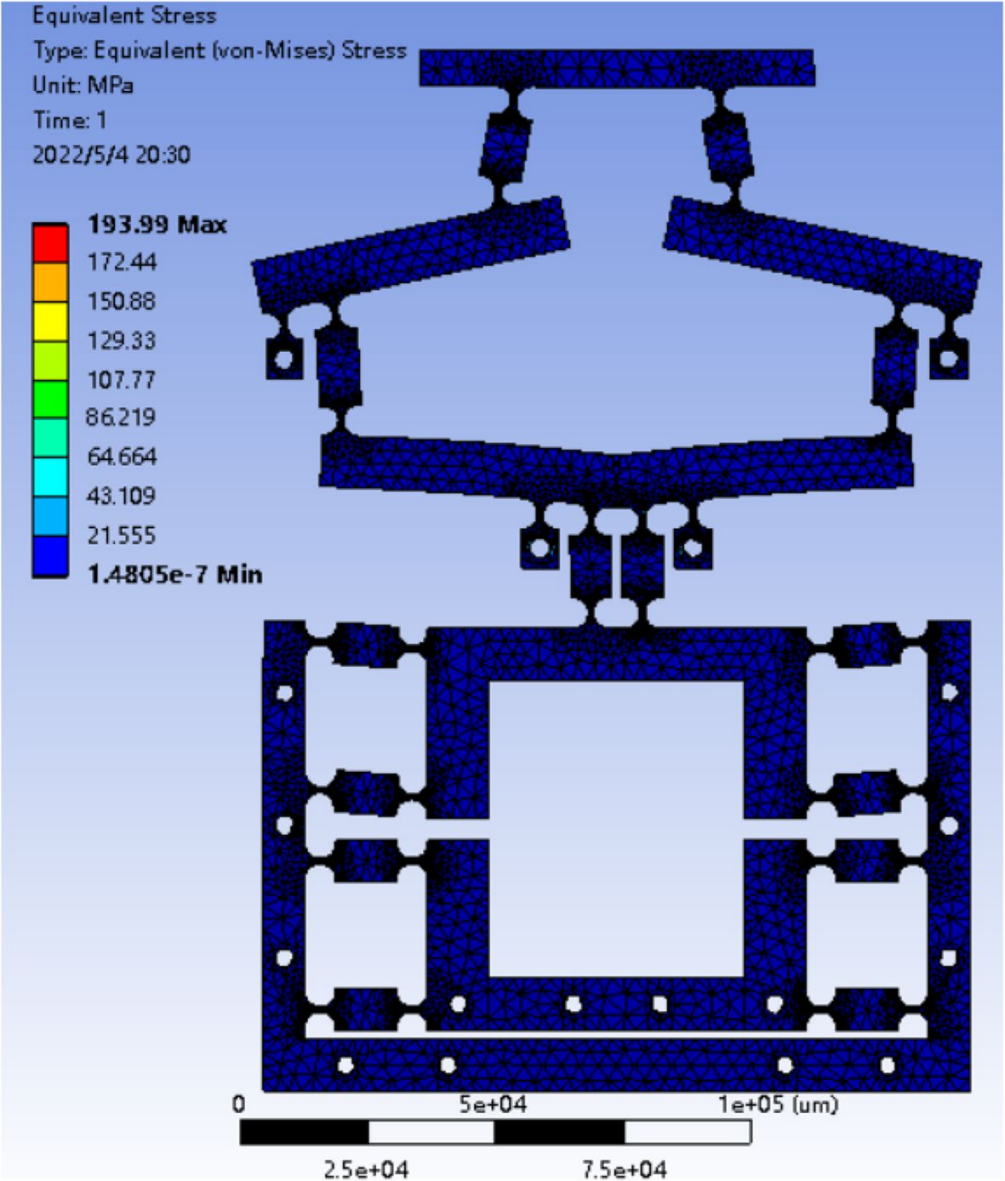
Stress cloud diagram of the micro-drive mechanism

The strength analysis of the micro-drive system mainly analyzes whether the micro-drive mechanism will be damaged under the driving of the micro-actuator. Therefore, it is necessary to analyze whether the micro-drive mechanism will be damaged under the maximum driving displacement of the PZT, that is, to analyze the maximum simulated stress of the micro-drive mechanism. The optimized micro-drive mechanism was imported into the ANSYS statics module, fixed constraints were imposed on the 18 bolt holes on the mechanism, and a negative Y-axis displacement of 15 μm was applied to the position of the actuator in the mechanism. As shown in Fig. 8, the maximum simulated stress of the mechanism was 193.99 MPa.

The allowable stress of the material is:

14 $$\left[\sigma \right]=\frac{\sigma_s}{\lambda }$$

The yield limit σ_s_ of 60Si2Mn material is 1176 MPa, and if the safety factor λ is set to 1.5, the allowable stress [σ] of the material will be 784 MPa. The maximum simulated stress of the mechanism was 193.99 MPa, which is much lower than that of the material. From the static finite element analysis, it can be seen that the mechanism is safe and reliable in the driving process of the PZT, and its maximum stress meets the requirements of material check strength. Therefore, the strength of the mechanism satisfied the design requirements.

### Analysis of motion performance

Motion performance is the most important performance metric of the system. In this study, the finite element method was used to analyze the motion performance of a micro-drive system. The preliminary treatment of finite elements was consistent with the preliminary treatment of the strength performance analysis. By changing the displacement conditions applied to the surface imprint, the probe function can be used on the upper surface of the mechanism to obtain different output displacements under different applied displacement conditions. The input displacement of the mechanism from 1 μm to 15 μm (the elongation range of the PZT ceramic actuator) was taken as the initial conditions for the analysis. Subsequently the output displacement values of the system were separately calculated.

**Fig. 9:**
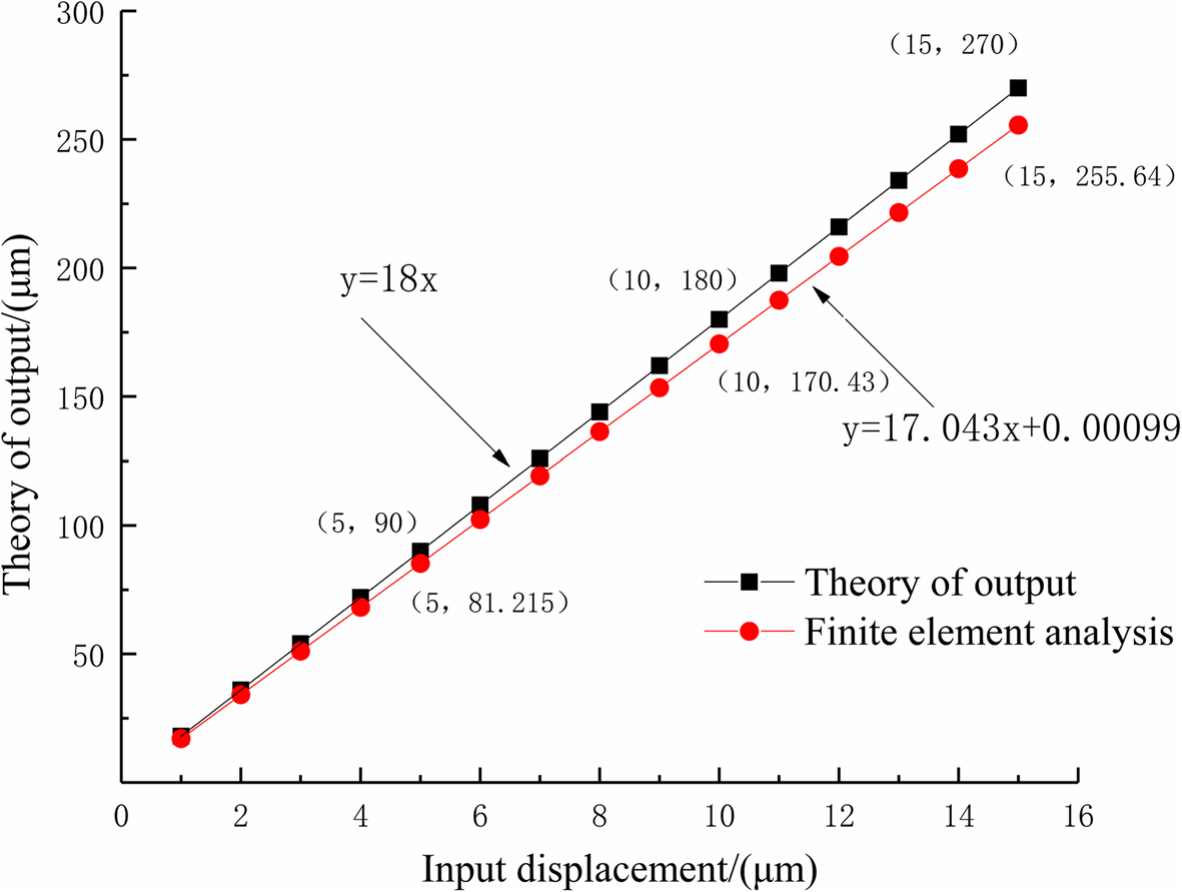
The linear fitting diagram of theoretical output and finite element analysis

The input value, theoretical output value, and finite element analysis results were linearly fitted, as shown in Fig. 9.

The linear fitting formula of the theoretical output for the optimized two-stage amplification micro-drive system is as follows:

15 $$\textrm{y}=18\textrm{x}$$

Its linearity is 1.

The linear fitting formula of the theoretical output for the optimized two-stage amplification micro-drive system is as follows:

16 $$\textrm{y}=17.043\textrm{x}+0.00099$$

Its linearity is 1.

The motion analysis shows that the maximum outputs of the theoretical calculation and finite element simulation are 270 μm and 255.64 μm respectively. The theoretical amplification ratio of the system and finite element simulation amplification ratio were 18 and 17.043, respectively, and the relative error was 5.31%. In ref. [17], the motion error of the micro-drive system was 7.89%, and the system proposed in this study had higher accuracy. Therefore, the system has the advantages of a large motion amplification ratio and high motion precision.

### Dynamic performance analysis

Inherent frequency is an important indicator for micro-drive mechanism dynamic performance evaluation; the higher the natural frequency of the micro-drive mechanism institutions the stronger the ability to resist vibration. Therefore, it is necessary to analyze the micro-drive mechanism using the modal analysis module of the finite element simulation software. The free modal analysis theory was chosen in this study because it can theoretically represent all vibration models of the micro-drive mechanism.

**Fig. 10:**
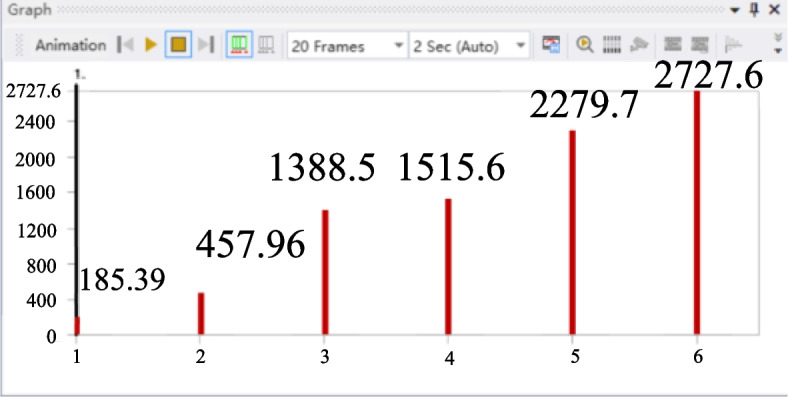
Diagram of the first six natural frequencies of the micro-drive mechanism

The preliminary treatment of finite element is consistent with that of strength performance analysis. The “. x_t “ file of the micro-drive mechanism was imported into the Modal analysis module. After setting the material and grid division, the first six order natural frequencies were obtained, as shown in Fig. 10.

According to the finite element analysis the first six natural frequencies of the micro-drive mechanism are 185.39 Hz, 457.96 Hz, 1388.5 Hz, 1515.6 Hz, 2279.7 Hz, and 2727.6 Hz respectively. The piezoelectric ceramic brake used in this study has a frequency of 300 Hz; therefore, there is no resonance phenomenon, and the dynamic performance of the two-stage amplification micro-drive system is excellent.

## Conclusions

Based on the problems of low motion accuracy, poor safety, and a limited amplification ratio of the current two-stage amplification micro-drive system, a two-stage precision amplification micromotion system was designed in this study. The system has a symmetrical structure of flexible hinge components, and the guiding function can eliminate parasitic motion and forces in non-motion directions to ensure the accuracy and safety of the system movement. Through an experiment on the driving performance of the PZT ceramic brake, it was verified that the driving performance of the micro-actuator was excellent and met the design requirements.

The structure of the micro-drive mechanism was optimized based on PSO, which achieves the goal of obtaining the maximum amplification ratio in a limited space. In addition, the maximum amplification ratio was increased from 5 to 18 times (the range of exercise was increased by 240%). The finite element method was used to analyze the performance of the optimized two-stage amplifying micro-drive system. The results show that the strength, motion, and dynamic performances of the system are excellent, and the motion accuracy is high, which meets the design requirements.

In the future, we will consider further optimization of the micro-drive system from different directions and verify the correctness and applicability of the optimized micro-drive system through experiments to provide a reference value for the research and design of micro-drive systems.

## Data Availability

Data related to the current study are available from the corresponding author upon reasonable request.

## Abbreviations

PSO: Particle swarm optimization
MEMS: Micro-electro-mechanical system
PZT: Piezoelectric ceramic actuator
